# Gene Expression Profile in Immortalized Human Periodontal Ligament Fibroblasts Through hTERT Ectopic Expression: Transcriptome and Bioinformatic Analysis

**DOI:** 10.3389/fmolb.2021.679548

**Published:** 2021-06-01

**Authors:** Lygia S. Nogueira, Carolina P. Vasconcelos, Geovanni Pereira Mitre, Leonardo Oliveira Bittencourt, Jessica Rodrigues Plaça, Maria Sueli da Silva Kataoka, João de Jesus Viana Pinheiro, Gustavo Pompermaier Garlet, Edivaldo H. C. De Oliveira, Rafael R. Lima

**Affiliations:** ^1^ Laboratory of Functional and Structural Biology, Institute of Biological Sciences, Federal University of Pará, Belém, Brazil; ^2^ Laboratory of Cell Culture and Cytogenetics, Environment Section, Evandro Chagas Institute, Ananindeua, Brazil; ^3^ School of Dentistry, Federal University of Pará, Belém, Brazil; ^4^ Regional Blood Center at University Hospital of the Ribeirão Preto Medical School of University of São Paulo, Ribeirão Preto, Brazil; ^5^ School of Dentistry of Bauru, University of São Paulo, Bauru, Brazil

**Keywords:** S100A7, cell culture, periodontal fibroblast, immortalization, hTERT

## Abstract

Human periodontal ligament fibroblast (hPLF) cells play an important role in maintaining oral cavity homeostasis with special function in tissue regeneration and maintenance of dental alveoli. Although their primary cell cultures are considered a good experimental model with no genetic changes, the finite life span may limit some experimental designs. The immortalization process increases cell life span but may cause genetic changes and chromosomal instability, resulting in direct effects on physiological cell responses. In this way, we aimed to investigate the global gene expression of hPLFs after the immortalization process by the ectopic expression of the catalytic subunit of the enzyme telomerase reverse transcriptase (hTERT) through transcriptome analysis. The embryonic origin of the primary culture of hPLF cells and immortalized hPLF-hTERT was also tested by vimentin staining, hTERT synthesis evaluated by indirect immunocytochemistry, analysis of cell proliferation, and morphology. The results indicated that hPLFs and hPLF-hTERT were positive for vimentin. On the 20th cell passage, hPLFs were in senescence, while hPLF-hTERT maintained their proliferation and morphology characteristics. At the same passage, hPLF-hTERT presented a significant increase in hTERT synthesis, but transcriptome did not reveal overexpression of the hTERT gene. Fifty-eight genes had their expression altered (11 upregulated and 47 downregulated) with the absence of changes in the key genes related to these cell types and in the main cancer-associated genes. In addition, the increase in hTERT protein expression without the overexpression of its gene indicates posttranscriptional level regulation. Successful immortalization of hPLFs through the ectopic expression of hTERT encourages further studies to design experimental protocols to investigate clinical questions from a translational perspective.

## Introduction

Human periodontal ligament fibroblast (hPLF) cells play an important role in oral cavity homeostasis, being an important contributor to the regeneration of the periodontium through the production and secretion of extracellular matrix components, especially collagen fibers, that link the alveolar bone to the cementum covering the tooth root (Marchesan et al., 2011; Smith et al., 2019). hPLF cells also participate actively in the immune and inflammatory events in periodontal diseases, producing cytokines and chemokines (Takashiba et al., 2003; Marchesan et al., 2011). These cells acquired from fresh periodontal ligament tissue are heterogeneous populations with different self-renewal capacities, wherein their potential for differentiation in a long-term culture can diminish the number of cells. Lallier and Spencer (2007) indicated that hPLF differentiation in culture was under the complex regulation of several soluble factors that possibly consigned these cells to distinct fates, suggesting that periodontal fibroblast culture is similar to fresh ligament tissue but especially represents the immature form of these cells.

*In vitro* experiments using isolated cells from fresh tissues usually reflect the biochemical responses of the cells *in vivo* but have the important restriction of limited life span and cells become senescent. Primary hPLF cultures demonstrated that senescence significantly impairs the ability toward an osteoblastic differentiation (Konstantonis et al., 2013). On the other hand, the continuous cell lines are easy to use, and have unlimited life span that provides homogeneous and reproducible data, and considered as transformed cells. This process generates either naturally or by genetic transformation named cell line immortalization.

There are several ways to acquire transformed cells but these mostly involve viruses such as Epstein–Barr virus (EBV), adenovirus, human papillomavirus (HPV), and simian virus 40 large T (SV40). SV40 protocol is one of the most used immortalizing agents, but their methodology is restricted by slowing of cellular growth and widespread apoptosis (Bryan and Reddel, 1994; Lustig, 1999). Evidence shows that the timing of cellular senescence in human cells is directly related to the length of telomeres and repetitive TTAGGG sequences at the ends of each chromosome (Cong et al., 2002), and most somatic cells undergo a progressive loss of their telomeric DNA in each division because of the end replication problem and other cellular/molecular events (Counter et al., 1998). In this perspective, the ectopic expression of the catalytic subunit of telomerase reverse transcriptase enzyme (hTERT) can successfully reverse this process.

Although previous studies have reported successful immortalization of the periodontal fibroblast cells by hTERT expression or the SV40 protocol (12–14), they focused on maintenance (or not) of the genes specifically related to the key functions of these types of cells. Thus, our main goal was to examine the overall gene expression profile of the immortalized human periodontal ligament fibroblast (hPLF-hTERT). We conducted the immortalization process in hPLF primary cells using the hTERT expression protocol, followed by confirmation of the immortalization process through indirect immunofluorescence of hTERT synthesis, cell proliferation, and cell morphology, and finally, we performed the gene expression profile through transcriptome analysis.

## Materials and Methods

### Ethics and Cell Line Immortalization

**FIGURE 1:**
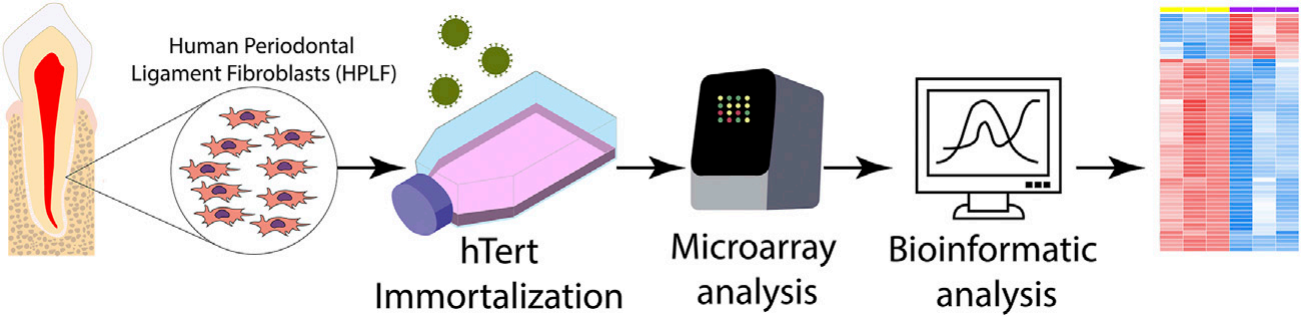
Methodological scheme of the experimental design. The primary culture of human periodontal ligament fibroblasts (hPLF) was immortalized by the telomerase reverse transcriptase enzyme (hTERT) protocol, and then the transcriptome analysis through microarray was performed using bioinformatics tools.

Primary hPLFs were obtained from human patients under approval of the Human Research Ethics Committee (Comitê de ética em pesquisa em seres humanos CEP-ICS/UFPA, CAEE number 0121.0.073.000-11). hPLFs from the primary culture were grown in two culture flasks, denominated F1 and F2, and kept in an incubator at 37°C in a humid atmosphere and 5% CO_2_. Concomitantly, in the F2 flask, only the cell culture medium was renewed. Hexadimethrine bromide (polybrene, Sigma-Aldrich^®^, St. Louis, MO, United States) was added to both flasks, and the immortalized strain was selected with antibiotic G428 (Sigma-Aldrich^®^). After 7 days, the selection was completed. All experimental steps are described in Figure 1.

### Maintenance of the Cell Culture

Both original hPLFs and immortalized hPLF-hTERT were cultured in Dulbecco’s modified Eagle’s medium (DMEM) and Ham’s F-12 nutrient medium (1:1), supplemented with 10% fetal bovine serum (FBS), 100 U/ml penicillin, and 100 μg/ml streptomycin, incubated at 37°C in 5% CO_2_ (Nogueira et al., 2019). The medium was changed every 48 h. When cells became fully confluent, they were passaged using 0.25% trypsin solutions and seeded in new flasks. About 20 passages were used in our experiments.

### Indirect Immunofluorescence

#### Cell Characterization

Primary cell culture (hPLF) and immortalized (hPLF-hTERT) cells were characterized using vimentin label (Costa et al., 2020). Cells were cultured on glass coverslips in 24-well plates, and then they were fixed in 2% paraformaldehyde for 10 min with 0.5% Triton x-100 solution (Sigma-Aldrich^®^; 15 min). Following that, cells were washed with PBS, incubated with 1% PBS/FBS (BSA, bovine serum albumin, Sigma-Aldrich^®^) for 30 min, and then incubated with the primary monoclonal antibodies diluted in 1% PBS/BSA for at least 12 h and at most 18 h in a humid chamber at 4°C. The primary antibody used was anti-vimentin, mouse monoclonal antibodies (1:100; Diagnostic BioSystems, Pleasanton, CA, United States). To detect primary antibodies, secondary antibodies conjugated to Alexa Fluor 488 or 588 (Invitrogen, Carlsbad, CA, United States) were used and Hoechst 33258 (1: 2000, Sigma, St. Louis, MO, United States) to label the nuclei. The secondary antibody and Hoechst were diluted in PBS/BSA and incubated for 1 h in a humid and dark chamber at room temperature. After that, the coverslip was washed for 5 min with PBS solution and twice in distilled water before being mounted on glass slides, using ProLong Gold antifade reagent (Invitrogen, Carlsbad, CA, United States). Afterward, the slides were examined under a fluorescence microscope (Axio Scope A1, Zeiss) equipped with a digital camera (AxioCam MRc, Zeiss). As a negative control, the same protocol was performed without incubation of the primary antibody.

#### Telomerase Reverse Transcriptase Enzyme Synthesis

The same protocol mentioned before was applied to evaluate the hTERT protein expression in the primary cell culture (hPLF) and immortalized cells (hPLF-hTERT). However, the cells were incubated with primary monoclonal antibody anti-TERT (1:50) diluted in 1% PBS/BSA for 12–18 h in a humid chamber at 4°C.

### Gene Expression

#### RNA Extraction

hPLF and hPLF-hTERT from 20a passage were maintained in cell culture conditions until they acquired 80% confluency. Following that, cells were removed using 0.25% trypsin solution and centrifuged (1,800 rpm for 5 min). The pellets formed in both flasks were used to extract the RNA using the SV total RNA isolation system kit from Promega^®^. The samples were homogenized in RNA lysis buffer containing beta-mercaptoethanol, followed by transfer to a fresh tube containing RNA dilution buffer. Samples were centrifuged and transferred to a new tube containing 95% ethanol solution. Following that, samples were transferred to a spin column, and three centrifugation steps of 1 min/14 *g* were performed. The first step used RNA wash solution, then the DNAse Stop solution, and, finally, an RNA wash solution. The RNA extracted was eluted into an elution tube using 15 µl of nuclease-free water. RNA quantification was performed using TapeStation 4200 (Agilent Technologies), and the A260/280 ratio analyzed using Nanodrop ND-1000 UV-Vis spectrophotometer, version 3.2.1 (Supplementary Material). The purified RNA was stored at −80°C for posterior gene expression assay.

#### Gene Expression Analysis—Microarray

The microarray analysis was performed using “one-color microarray–based gene expression analysis” (Agilent technologies, EUA). The RNA obtained from hPLF and hPLF-hTERT extraction was used to synthesize the first cDNA, which occurs through reverse transcription assisted by T7 RNA polymerase. The cRNA was transcripted from the second cDNA strand, and 3-cyanine was labeled into the cRNA, followed by RNA purification. The RNA purification was performed using the RNeasy Mini Spin Kit. The cRNA was quantified for spectrophotometry, where the A260/280 ratio and concentration were also analyzed (Nanodrop ND-1000 UV-Vis, version 3.2.1). In order to perform the hybridization, the fragmentation mix was added to the RNA from both hPLF and hPLF-hTERT samples and incubated at 60°C for 30 min. Following that, to each sample 25 µl of 2x-RPM hybridization buffer was added at 4°C. Then 40 µl of each sample was added to the hybridization lamina and left in the hybridization camera for 17 h at 10 rpm and 65°C. After that, the samples were read in a microarray scanner from Agilent (G4900DA) and the images obtained from the software Feature Extraction v10.10 for data analysis through bioinformatics.

### Bioinformatic Analysis

Quality control, quantile normalization, and batch effect removal were performed using the limma package. Differentially expressed genes were identified based on an absolute log2-fold change level >1 and the *p*-value adjusted by FDR <0.05. Overrepresentation analysis for differently expressed genes of gene ontology (GO) terms or KEGG pathways was also done with the limma package. Overrepresented *p*-values were adjusted by the Bonferroni method, and only adjusted *p*-values <0.05 were considered.

## Results

### Human Periodontal Ligament Fibroblasts *Vs.* Immortalized Human Periodontal Ligament Fibroblasts

**FIGURE 2:**
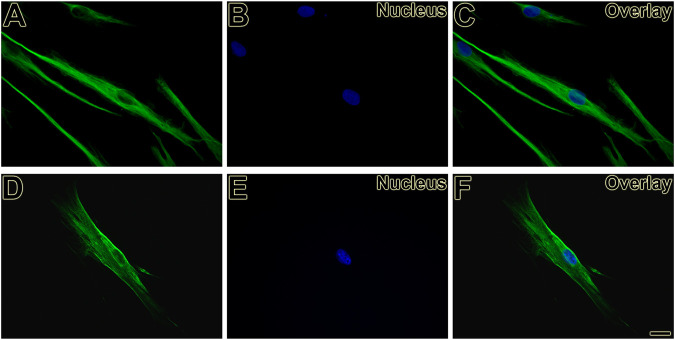
Vimentin expression present as filamentous network in hPLF **(A, C)** and hPLF-hTERT **(D, F)** demonstrating the mesenchymal origin in both cell lines. Hoechst 33258 was used for nuclear counterstaining **(B, C, E, F)**. Scale bar: 20 µm.

**FIGURE 3:**
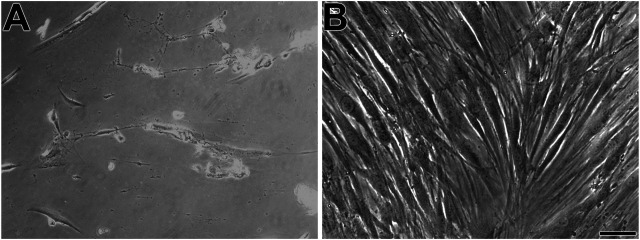
Phase-contrast microscopy displays hPLF **(A)** and immortalized human periodontal fibroblasts (hPLF-hTERT) **(B)**, both cell lines with spindle-shaped phenotype. Scale bar: 100 µm.

**FIGURE 4:**
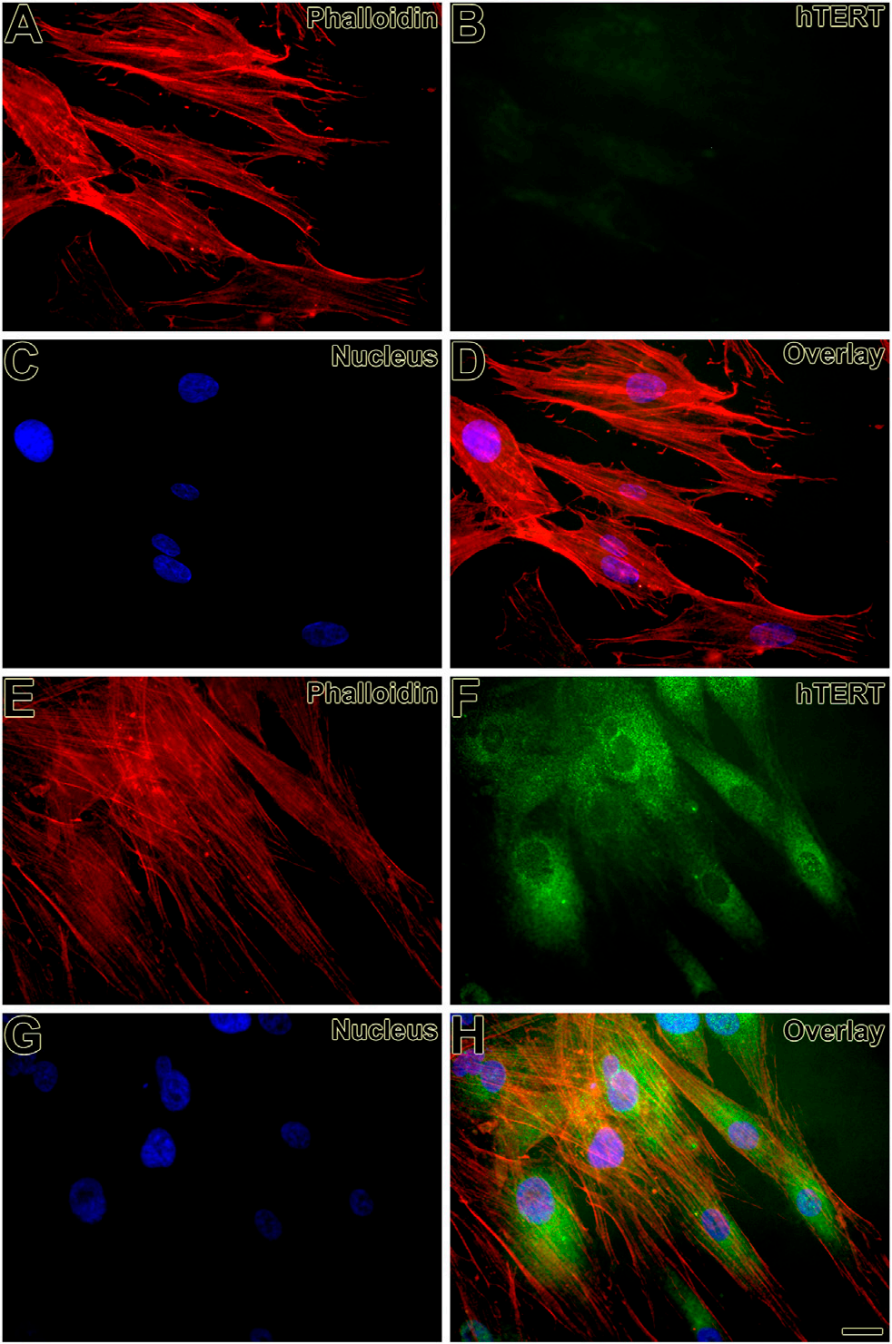
Expression of hTERT was weak in hPFL **(B, D)** and strong in hPLF-hTERT **(F, H)** cell lines, respectively, and observed as an intracellular dot-like staining. Actin staining (Alexa Fluor 568 phalloidin) reveals the cortical cytoskeleton **(A, D, E, H)**. Hoechst 33258 was used for nuclear counterstaining **(C, D, G, H)**. Scale bar: 20 µm.

First, the mesenchymal origin of the cell lines hPLF and hPLF-hTERT analyzed through indirect immunofluorescence was found to be positive for vimentin in both of them (Figures 2A–F). Following that, to initiate the evaluation of the immortalization performance, both cell lines were tested through antibiotic selection. As a result, phase-contrast photomicrography exhibited cell death in hPLF during the selection (Figure 3A), while hPLF-hTERT cells maintained proliferation and cell morphology (Figure 3B). Last, the performance of indirect immunofluorescence to evaluate the expression of hTERT after immortalization demonstrated weak hTERT staining in hPLF, while hPLF-hTERT showed an expressive presence of the protein. In terms of percentage, the fluorescence of phalloidin, hTERT, and nucleus represents 73.61, 17.48, and 8.89%, respectively, while in the immortalized hPLF-hTERT cells, these percentages were 43.89, 47.0, and 8.98%, respectively (Figure 4).

### Immortalization Process Modulated the Expression of a Few Genes

**FIGURE 5:**
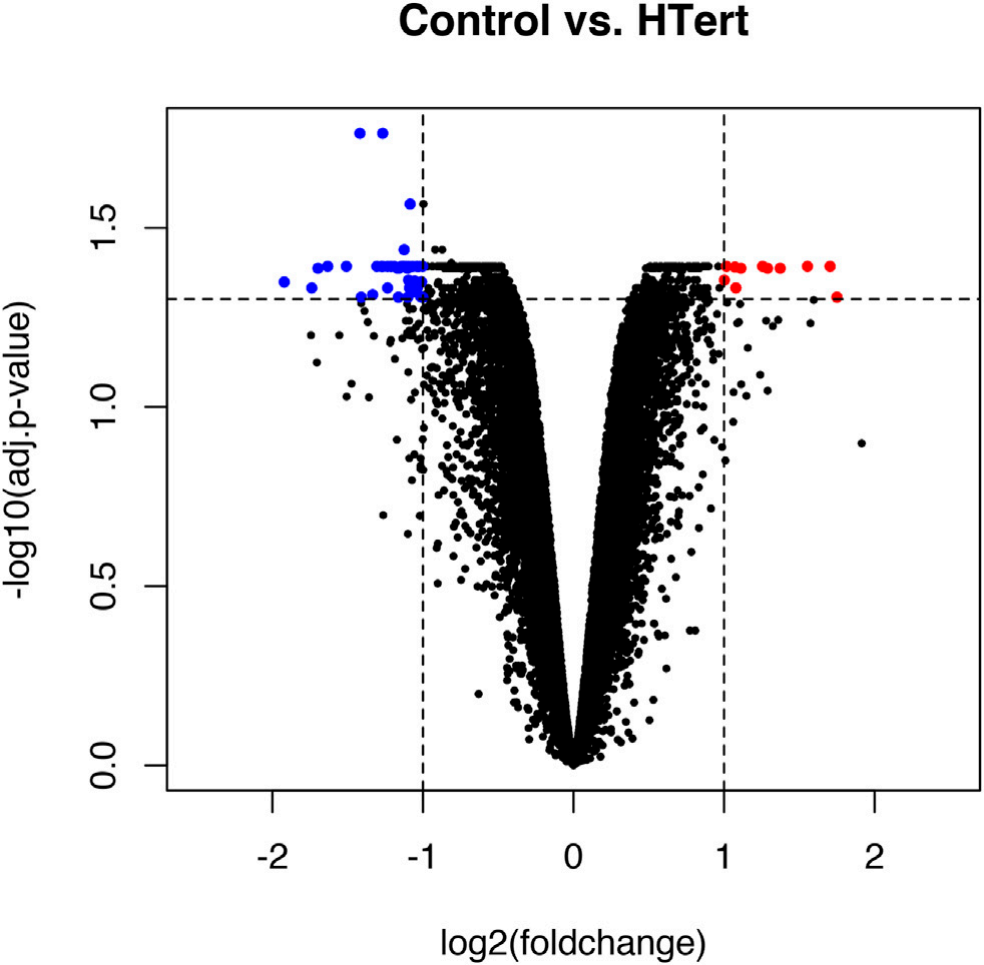
Volcano plot representing the global profile of gene expression of hPLF cells after the immortalization procedure. Blue dots represent downregulated genes and red dots represent upregulated genes. Data were expressed as log2FC > 1 or < −1 and analyzed by Student’s *t*-test, adopting adj. *p* < 0.05 (*n* = 3/each group).

**FIGURE 6:**
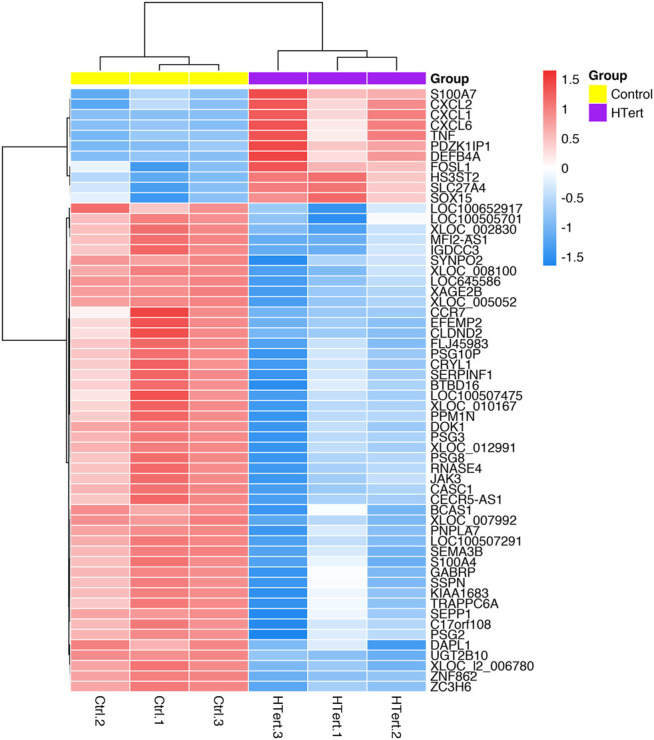
Heatmap of differentially expressed genes in hPLF after the immortalization process (control vs. hTERT). The dendrograms represent the hierarchical cluster relationship between genes (left side) and among samples (upper), based on Pearson’s correlation; log2FC > 1 or < −1. log2-fold change >1.5 or <−1.5; adj.; cut-off adj. *p*-value: <0.05.

Fifty-eight genes were found to be differentially expressed, in which 11 were upregulated and 47 downregulated (Figure 5; Table 1). In terms of percentage, the altered genes after the immortalization process represent 0.22% of the total 26,000 genes analyzed through the microarray. As observed in the heatmap (Figure 6), along with the 58 genes, there was a pattern of gene expression modulation among each group of cells, represented by the proximity of dendrograms.

**TABLE 1:** Top ten genes upregulated and downregulated showing altered expression in hPLF-hTERT compared to the primary cell culture of hPLF. Gene fold regulation and *p*-value are reported for each gene.

Gene symbol	Gene description	logFC (x10^14^)	Adjusted *p*-value
FOSL1	FOS like 1, AP-1 transcription factor subunit	1.75	0.049
CXCL1	C-X-C motif chemokine ligand 1	1.70	0.040
S100A7	S100 calcium-binding protein A7	1.55	0.040
DEFB4A	Defensin beta 4A	1.37	0.040
PDZK1IP1	PDZK1 interacting protein 1	1.26	0.040
CXCL2	C-X-C motif chemokine ligand 2	1.11	0.040
SOX15	SRY-box transcription factor 15	1.08	0.046
SLC27A4	Solute carrier family 27 member 4	1.07	0.040
HS3ST2	Heparan sulfate-glucosamine 3-sulfotransferase 2	1.02	0.040
CXCL6	C-X-C motif chemokine ligand 6	1.29	0.040
XAGE2B	X antigen family member 2	−1.28	0.040
PNPLA7	Patatin-like phospholipase domain–containing 7	−1.31	0.040
LOC100505701	Uncharacterized	−1.33	0.048
BTBD16	BTB domain–containing 16	−1.41	0.049
ZNF862	Zinc finger protein 862	−1.42	0.017
CECR5-AS1	HDHD5 antisense RNA 1	−1.51	0.040
IGDCC3	Immunoglobulin superfamily DCC subclass member 3	−1.63	0.040
C17orf108	LYR motif–containing 9	−1.70	0.040
SSPN	Sarcospan	−1.74	0.046
GABRP	Gamma-aminobutyric acid type a receptor subunit pi	−1.92	0.0447

**FIGURE 7:**
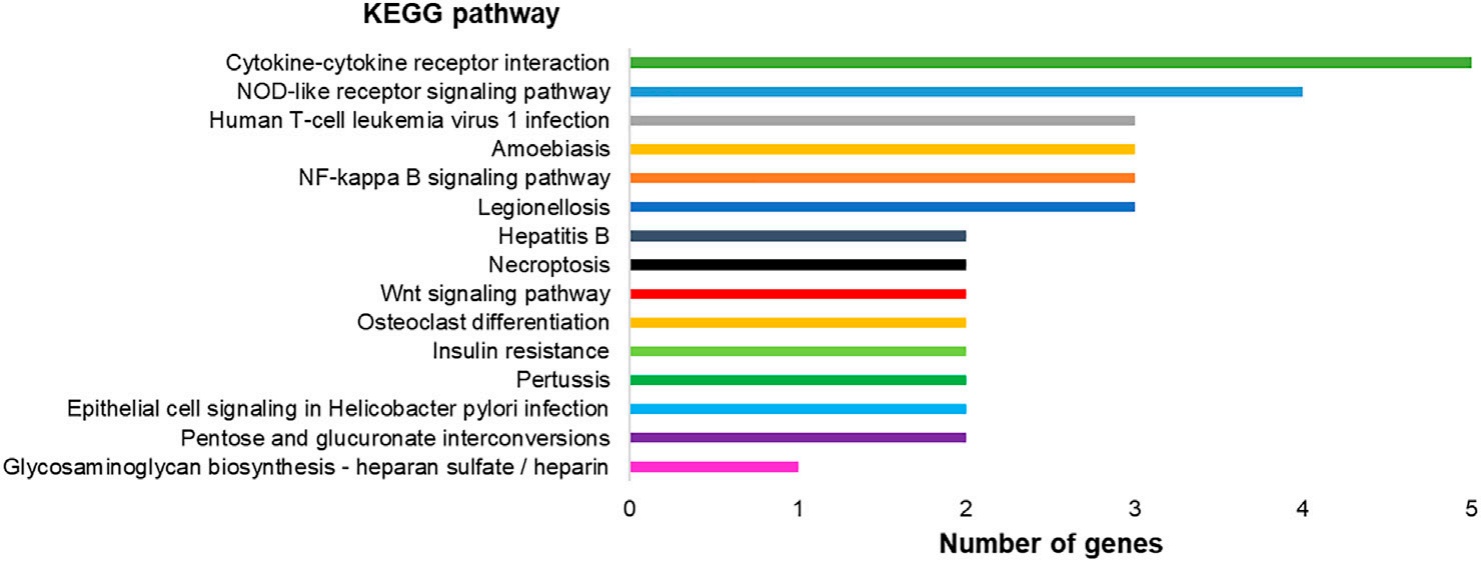
Graph representing KEGG pathways associated with the differentially expressed genes in hPLF after immortalization processes and their respective number of genes found in the transcriptome analysis.

Modulated genes are involved in 20 different pathways, as observed in Figure 7, including TNF signaling pathway (four genes), NOD-like receptor signaling pathway (four genes), NF-kappa B signaling pathway (three genes), amebiasis (three genes), human T-cell leukemia virus 1 infection (three genes), cytokine–cytokine receptor interaction, and 15 other pathways.

### Analysis of Gene Ontology of the Transcriptome

**FIGURE 8:**
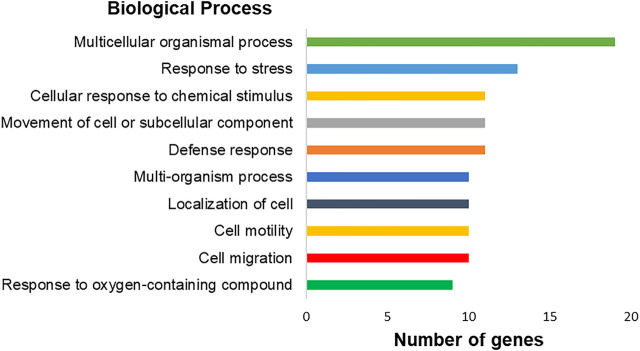
Graph representing the top-ranking biological processes of differentially expressed genes in hPLF after immortalization processes and their respective number of genes found by transcriptome analysis. Biological processes based on gene ontology annotation, adopting *p* < 0.05.

The genes with significantly altered expression are involved in 447 different biological processes. Among them, we highlighted ten processes with the highest number of involved genes: a multicellular organismal process (17 genes), extracellular region (17 genes), response to external stimulus (14 genes), extracellular space (13 genes), extracellular region part (13 genes), response to stress (13 genes), locomotion (12 genes), defense response (11 genes), movement of cell or subcellular component (11 genes), and cellular response to chemical stimulus (11 genes) (Figure 8). Besides that, changes in biological processes related specifically to the immortalization process, such as cell migration, cell motility, and cell adhesion, were observed.

## Discussion

In this study, immortalization of the primary cell culture of the periodontal ligament through the hTERT technique helped reach the goal of increasing the life span of cells through the maintenance of proliferation and morphology. Surprisingly, the process was posttranscriptionally controlled by the significant increase of hTERT synthesis with no change in hTERT gene expression. The identification of 58 changes in the gene profile after hPLF cell immortalization is considered a low number compared with changes in gene expression of fresh tissue and hPLF after culturing (Lallier and Spencer, 2007). In the study published by Lallier and Spencer (2007), although these populations were considered similar, they differed in their expression of roughly 3,240 genes (6% of total analyzed). Thus, considering only the gene numbers, the hTERT immortalization protocol performed by us did not generate profound gene modifications ,which is in agreement with the observations of low phenotypic and karyotypic changes of this protocol compared with a virus protocol, such as SV40 (Wang et al., 2019).

The first indication of a successful immortalization process is hPLF-hTERT cultivation over the 20th passage. The primary cell culture of hPLF showed characteristics of senescence (Chen et al., 2000) at the 20th passage, with a large and flattened morphology and decreased rate of proliferation, while the hPLF-hTERT maintained their fusiform shape and short cytoplasmic extensions, preserving their proliferation and normal morphology.

The second indicator of a functional immortalization process occurred from labeling the hTERT expression using indirect immunocytochemistry. The immortalized cells, hPLF-hTERT, exhibited an expressive increase in telomerase activity/expression, which is considered one of the major biochemical steps to increase cell culture life span, but not sufficient *per se* for oncogenic transformations (Franzese et al., 2002), while the primary cell culture of hPLF demonstrated a weak labeling expression of the catalytic subunit hTERT, consistent with most human cells that are telomerase negative (Weinrich et al., 1997).

The hTERT telomerase expression was estimated to occur transcriptionally in immortalized hPLF-hTERT cells, but upregulation of the hTERT gene did not occur. As observed for other cell types such as lymphocytes (Liu et al., 2001) and HTLV-I cells (Bellon and Nicot, 2015), the transcriptome result leads us to believe that the hTERT regulation was carried out in a posttranscriptional manner. According to the literature, nuclear factor-kB (NF-kB) is the main key in the regulation of telomerase activity through the modulation of nuclear translocation that occurs mainly over the induction of the tumor necrosis factor (TNF) (Akiyama et al., 2003), which is one of the most potent physiological inducers of the nuclear transcription factor NF-kB (Liu et al., 2017). In our study, upregulation of TNF in immortalized hPLF-hTERT cells, along with the observed changes in the NF-kβ pathway, highly indicates the posttranscriptional process.

In addition to this mechanism, an important marker of hTERT dysfunction was upregulated in hPLF-hTERT, the S100A7 gene. The occurrence of upregulated S100A7 is a direct consequence of telomere dysfunction in human keratinocytes independently of cell cycle regulators (de Castro et al., 2014). Besides that, the overexpression of S100A7 is associated with a chemokine and cytokine action (Jinquan et al., 1996; D’Amico et al., 2016), involved in an inflammatory response and latter stimuli to odontoclast differentiation (Charoenpong et al., 2019).

Although we did not observe an overexpression of the hTERT gene, there are markers and pathways that explain the observed successful cell immortalization of the hPLF primary cell culture. Based on gene ontology results, important characteristics of the overexpression of hTERT in other cell types, such as promotion of cell adhesion, motility, and migration (Chen et al., 2013; Liu et al., 2016), were also observed in the hPLF-hTERT. Altered genes were related to cell motility (GO: 0048870 = 10 genes) and regulation of cell motility (GO: 2000145 = 5 genes), cell migration (GO: 0016477 = 10 genes), positive regulation of heterotypic cell–cell adhesion (GO: 0034116 = 1 gene), regulation of cell–cell adhesion (GO: 0022407 = 3 genes), and positive regulation of cell adhesion (GO: 0045785 = 3 genes).

Taking into account the pathways designated as altered by KEGG (Kyoto Encyclopedia of Genes and Genomes) in hPLF-hTERT, most parts of them are exclusively linked to pro-inflammatory response, innate immune response, and metabolism responses. The fibroblasts of the periodontal ligament participate in immune and inflammatory events in periodontal diseases, with producing cytokines and chemokines as one of their main functions (Takashiba et al., 2003), besides being responsible for structural composition and secretion of extracellular components (e.g., collagen and glycosaminoglycans) and tissue repair (Marchesan et al., 2011). However, the immortalization process did not cause direct changes in genes related to mineralization (osteopontin, alkaline phosphatase, osteomodulin, and collagen type I, II, and V), cell motility (collagen type I and fibronectin), and cell migration (collagen type 1) and proliferation, thus implicating its importance in the maintenance of hPLF characteristics and main functions. These observations agree with previous studies where the immortalization process (by hTERT expression or SV40 protocol) induced the increase in life span without cells losing their ability to repair and regenerate dental and periodontal tissues (Kamata et al., 2004; Fujii et al., 2006; Tomokiyo et al., 2008).

One of the altered pathways in hPLF-hTERT with the highest number of genes overexpressed was the cytokinine–cytokine receptor pathway. Cytokines represent a diverse group of molecules that bind to receptors on target cells and activate a cascade of intercellular signals. One of the families that belong to this pathway is the CXCL family; three of their genes were upregulated in our study (CXCL1, CXCL4, and CXCL6). In the oral cavity, the role of cytokines in the progression of periodontitis is particularly important as they act as the first response against pathogens and connect tissue cells with lymphocytes and accessory cell populations (Graves, 2008).

The most overexpressed gene in hPLF-hTERT was FOSL1 or AP-1 which is linked by different stimuli, such as inflammatory cytokines, stress inducers, or pathogens, resulting in innate and adaptive immunity (Gazon et al., 2017). AP-1 is also involved in various cellular events, including differentiation, proliferation, survival, and apoptosis. The downregulation causes inhibition of growth cell lines and tumor both *in vivo* and *in vitro* (Eckert et al., 2013; Kharman-Biz et al., 2013). In hPLF-hTERT, this gene is related to different pathways pointed out by KEGG, such as epithelial cell signaling in *Helicobacter pylori* infection, pertussis, hepatitis B, TNF signaling pathway, and NOD-like receptor signaling pathway.

Another important upregulated gene in hPLF-hTERT was beta-defensin 2 (DEFB4A). Two of several different pathways of gene activation were observed in the immortalized cell line: NOD-2–dependent NF-kβ activation (Wang et al., 2019). Crucial in the innate immune response (Saxena and Yeretssian, 2014), the NOD-like receptors (NLRs) in signaling pathways are transmembrane receptors that show various functions such as inflammasome formation, signaling transduction, transcription activation, and autophagy (Kim et al., 2016). The increase in beta-defensin 2 protein is considered pro-inflammatory and innate immune response, which has marked antimicrobial properties (Roesner et al., 2017).

## Conclusion

Taking together, the immortalization process using ectopic expression of hTERT in the hPLF primary cell culture resulted in an increase in life span of cells with the maintenance of their phenotypic characteristics. Although some studies suggest the requirement of secondary inactivation of regulator pathways, such as p16 and pRB, for the cell immortalization process (Counter et al., 1998; Wall et al., 2016), our study is in agreement with others and suggests the reconstitution of telomerase by hTERT activation in a posttranscriptional manner that is sufficient to immortalize primary human cells. These results indicate in a pioneering way the changes that occurred in the general profile of hPLF gene expression after the immortalization process. In the future, hPLF-hTERT should be evaluated regarding the maintenance of its physiological responses to periodontal ligament fibroblasts, such as collagen production, extracellular matrix, and inflammatory responses. Thus, new studies need to be developed in order to create experimental protocols that investigate clinical issues from a translational perspective.

## Data Availability

The original contributions presented in the study are included in the article/Supplementary Material. Further inquiries can be directed to the corresponding author.
