# Supplementary material for: Gene Expression Profile in Immortalized Human Periodontal Ligament Fibroblasts Through hTERT Ectopic Expression: Transcriptome and Bioinformatic Analysis
Source: Front Mol Biosci. 2021 Jun 1;8:679548. doi: 10.3389/fmolb.2021.679548 (PMC8204186; doi:10.3389/fmolb.2021.679548)
Supplement: Supplementary file 1 [file DataSheet1.docx]

**Supplementary material**

List of 50 genes showing altered expression in immortalized human periodontal fibroblasts (hPLF-hTERT) compared to the primary cell culture of human periodontal fibroblasts (hPLF). Gene fold regulation and P-value is reported for each gene.

| Gene symbol | Gene description | logFC (x10^14^) | Adjusted P-value |
| --- | --- | --- | --- |
| FOSL1 | FOS Like 1, AP-1 Transcription Factor Subunit | 1.75 | 0.049 |
| CXCL1 | C-X-C Motif Chemokine Ligand 1 | 1.70 | 0.040 |
| S100A7 | S100 Calcium Binding Protein A7 | 1.55 | 0.040 |
| DEFB4A | Defensin Beta 4A | 1.37 | 0.040 |
| PDZK1IP1 | PDZK1 Interacting Protein 1 | 1.26 | 0.040 |
| CXCL2 | C-X-C Motif Chemokine Ligand 2 | 1.11 | 0.040 |
| SOX15 | SRY-Box Transcription Factor 15 | 1.08 | 0.046 |
| SLC27A4 | Solute Carrier Family 27 Member 4 | 1.07 | 0.040 |
| HS3ST2 | Heparan Sulfate-Glucosamine 3-Sulfotransferase 2 | 1.02 | 0.040 |
| CXCL6 | C-X-C Motif Chemokine Ligand 6 | 1.29 | 0.040 |
| TNF | Tumor Necrosis Factor | 1.00 | 0.044 |
| XLOC_007992 |  | -1.00 | 0.040 |
| LOC100507475 |  | -1.00 | 0.049 |
| PPM1N | Protein Phosphatase, Mg2+/Mn2+ Dependent 1N (Putative) | -1.01 | 0.044 |
| S100A4 | S100 Calcium Binding Protein A4 | -1.01 | 0.044 |
| PSG2 | Pregnancy Specific Beta-1-Glycoprotein 2 | -1.02 | 0.049 |
| CASC1 | Cancer Susceptibility 1 | -1.02 | 0.040 |
| BCAS1 | Breast Carcinoma Amplified Sequence 1 | -1.04 | 0.046 |
| EFEMP2 | EGF Containing Fibulin Extracellular Matrix Protein 2 | -1.04 | 0.040 |
| XLOC_002830 |  | -1.04 | 0.040 |
| SERPINF1 | Serpin Family F Member 1 | -1.06 | 0.047 |
| LOC100652917 |  | -1.06 | 0.044 |
| RNASE4 | Ribonuclease A Family Member 4 | -1.07 | 0.040 |
| KIAA1683 | IQ Motif Containing N | -1.09 | 0.046 |
| XLOC_l2_006780 |  | -1.09 | 0.027 |
| TRAPPC6A | Trafficking Protein Particle Complex 6A | -1.10 | 0.049 |
| XLOC_010167 |  | -1.10 | 0.044 |
| CLDND2 | Claudin Domain Containing 2 | -1.10 | 0.040 |
| XLOC_012991 |  | -1.10 | 0.040 |
| DOK1 | Docking Protein 1 | -1.11 | 0.040 |
| JAK3 | **Janus Kinase 3** | -1.12 | 0.040 |
| ZC3H6 | Zinc Finger CCCH-Type Containing 6 | -1.12 | 0.036 |
| FLJ45983 | GATA3-AS1 | -1.13 | 0.040 |
| DAPL1 | Death Associated Protein Like 1 | -1.13 | 0.040 |
| MFI2-AS1 | MELTF Antisense RNA 1 | -1.13 | 0.040 |
| XLOC_005052 |  | -1.13 | 0.040 |
| PSG8 | Pregnancy Specific Beta-1-Glycoprotein 8 | -1.14 | 0.040 |
| LOC645586 | Uncharacterized | -1.14 | 0.040 |
| CCR7 | C-C Motif Chemokine Receptor 7 | -1.16 | 0.049 |
| PSG10P | Pregnancy Specific Beta-1-Glycoprotein 10, Pseudogene | -1.16 | 0.040 |
| CRYL1 | Crystallin Lambda 1 | -1.17 | 0.040 |
| PSG3 | Pregnancy Specific Beta-1-Glycoprotein 3 | -1.19 | 0.040 |
| LOC100507291 | Uncharacterized | -1.21 | 0.040 |
| SEMA3B | Semaphorin 3B | -1.22 | 0.040 |
| SYNPO2 | Synaptopodin 2 | -1.23 | 0.040 |
| SEPP1 | Selenoprotein P | -1.24 | 0.046 |
| XLOC_008100 |  | -1.27 | 0.040 |
| UGT2B10 | UDP Glucuronosyltransferase Family 2 Member B10 | -1.27 | 0.017 |
| XAGE2B | X Antigen Family Member 2 | -1.28 | 0.040 |
| PNPLA7 | Patatin Like Phospholipase Domain Containing 7 | -1.31 | 0.040 |
| LOC100505701 | Uncharacterized | -1.33 | 0.048 |
| BTBD16 | BTB Domain Containing 16 | -1.41 | 0.049 |
| ZNF862 | Zinc Finger Protein 862 | -1.42 | 0.017 |
| CECR5-AS1 | HDHD5 antisense RNA 1 | -1.51 | 0.040 |
| IGDCC3 | Immunoglobulin Superfamily DCC Subclass Member 3 | -1.63 | 0.040 |
| C17orf108 | LYR Motif Containing 9 | -1.70 | 0.040 |
| SSPN | Sarcospan | -1.74 | 0.046 |
| GABRP | Gamma-Aminobutyric Acid Type A Receptor Subunit Pi | -1.92 | 0.0447 |
